# A Multi-Target Localization and Vital Sign Detection Method Using Ultra-Wide Band Radar

**DOI:** 10.3390/s23135779

**Published:** 2023-06-21

**Authors:** Jingwen Zhang, Qingjie Qi, Huifeng Cheng, Lifeng Sun, Siyun Liu, Yue Wang, Xinlei Jia

**Affiliations:** Emergency Research Institute, Chinese Institute of Coal Science CICS, Beijing 100013, China; zhangjingwen@mail.ccri.ccteg.cn (J.Z.); chenghuifeng@mail.ccri.ccteg.cn (H.C.); sunlifeng_2005@126.com (L.S.); liusiyun1019@163.com (S.L.); 13331361815@163.com (Y.W.); ybyyfz@163.com (X.J.)

**Keywords:** ultra-wideband radar (UWB), life detection, permutation entropy (PE), cluster analysis, vital sign extraction, ensemble empirical mode decomposition (EEMD), stochastic resonance (SR)

## Abstract

Life detection technology using ultra-wideband (UWB) radar is a non-contact, active detection technology, which can be used to search for survivors in disaster rescues. The existing multi-target detection method based on UWB radar echo signals has low accuracy and has difficulty extracting breathing and heartbeat information at the same time. Therefore, this paper proposes a new multi-target localization and vital sign detection method using ultra-wide band radar. A target recognition and localization method based on permutation entropy (PE) and K means++ clustering is proposed to determine the number and position of targets in the environment. An adaptive denoising method for vital sign extraction based on ensemble empirical mode decomposition (EEMD) and wavelet analysis (WA) is proposed to reconstruct the breathing and heartbeat signals of human targets. A heartbeat frequency extraction method based on particle swarm optimization (PSO) and stochastic resonance (SR) is proposed to detect the heartbeat frequency of human targets. Experimental results show that the PE—K means++ method can successfully recognize and locate multiple human targets in the environment, and its average relative error is 1.83%. Using the EEMD–WA method can effectively filter the clutter signal, and the average relative error of the reconstructed respiratory signal frequency is 4.27%. The average relative error of heartbeat frequency detected by the PSO–SR method was 6.23%. The multi-target localization and vital sign detection method proposed in this paper can effectively recognize all human targets in the multi-target scene and provide their accurate location and vital signs information. This provides a theoretical basis for the technical system of emergency rescue and technical support for post-disaster rescue.

## 1. Introduction

In recent years, earthquakes, landslides, house collapse and other disasters occur frequently, which seriously endangers people’s lives, safety and property. The first task of disaster rescue is to search for, and rescue trapped people in order to minimize casualties. This places a very high demand on life detection technologies and equipment. The ultra-wideband (UWB) life detection radar is a kind of high-tech life-saving piece of equipment which combines micro-power UWB radar technology with biomedical engineering technology [1,2,3,4,5,6]. It has the advantages of strong penetration, high resolution, good anti-jamming etc. [7,8,9,10]. It emits nanosecond pulses of electromagnetic waves over long distances, based on the time-domain Doppler effect generated by human motion on radar echoes, to analyze the presence of human targets in the detection range and the specific location and vital signs of each target [11,12].

In terms of life detection technology research based on the UWB radar, many scholars have conducted research on single target positioning and vital sign information extraction methods and achieved good results [13,14,15]. Dou Mingwu proposed a ToA (Time of Arrival) estimation method based on the characteristics of vital signals, which can be used to estimate the distance between the human target and the radar under conditions of wall penetration [16]. Zhang Zhu proposed a breathing signal extraction algorithm for human targets buried in rubble [17]. Wu Shiyou simulated a slow-time signal including respiratory response as a stochastic process with zero-mean random noise, realizing quasi-real-time detection of human respiration [18]. Qi Qingjie proposed a method that combines strong physical sign information extraction at P time and Variational Mode Decomposition (VMD) to extract the breathing and heartbeat information [19]. However, when there are multiple human targets randomly distributed in the environment, due to the influence of noise and mutual interference between targets, the above methods are likely to cause poor recognition of targets, making it difficult to accurately extract the position and vital sign information of each target.

In terms of research on multi-target detection methods, Zhang Yang proposed a variety of methods such as time-frequency analysis and improved constant false alarm rate (CFAR) to recognize multiple human targets at different distances [20,21]. Some researchers used the VMD algorithm to detect multiple human targets located at the same distance in an indoor environment and provide breathing frequency information [22,23]. Liu proposed a signal processing algorithm based on source separation and empirical mode decomposition to recognize and locate two subjects buried at different depths, which was able to extract the breathing features of the subjects but failed to capture the heartbeat frequency information [24]. Ren proposed a phase algorithm based on the logarithmic method to extract the heartbeat frequency of multiple human targets, but the distance between the human target and the radar was not more than 1 m, which is not suitable for post-disaster rescue scenarios [25]. It can be seen that these researches mainly focus on multi-target positioning and breathing information extraction, and there are few methods referring to heartbeat frequency information extraction. In addition, the performance and scope of application of these detection algorithms need to be further improved.

In the actual multi-target detection scene, when multiple targets are close to each other, the near-end target is likely to interfere with other targets due to reasons such as shading or tailing, and it is impossible to effectively separate multiple targets, which may easily cause recognition omission of targets. When multiple targets are far apart, due to the attenuation effect, the signal of the remote target is weak, and it is easy to be submerged in the background noise, so that the human targets cannot be effectively identified and the vital sign information cannot be accurately extracted. Therefore, it is a relatively difficult task to accurately recognize and locate each target and simultaneously extract breathing and heartbeat information in a multi-target detection scene. This paper proposes a new method for multi-target localization and vital sign information detection based on UWB radar. Specifically, a life detection and location method based on permutation entropy and the K means++ clustering algorithm is proposed, which can effectively recognize human targets in the environment and provide accurate quantity and distance information. An adaptive denoising method based on ensemble empirical mode decomposition (EEMD) and wavelet analysis (WA) is proposed, which can adaptively filter out local feature signals that do not contain breathing and heartbeat information in the tested signal. A heartbeat frequency extraction method based on particle swarm optimization (PSO) and stochastic resonance (SR) is proposed, which can effectively extract the weak heartbeat information of trapped people in cases of a low signal-to-noise ratio.

This paper is organized as follows. In Section 2, the model for life detection is discussed. In Section 3, the proposed method is presented in detail. In Section 4, the UWB radar system used in the experiment and the design of the experimental scheme are introduced. Section 5 discusses and verifies the performance of the proposed method. Section 6 concludes this paper and presents prospects for future research.

## 2. Life Detection Model

When a radar wave penetrates a certain obstacle and meets the human body, the reflected echo signal is modulated by the micro-movement caused by human life activities (such as breathing and heartbeat), which makes some parameters of the echo signal change. After the echo signal is received coherently, a static target echo such as a wall is filtered and a moving target echo such as a heart or lung is detected, and the vital signs information related to the human body can be extracted from it. The theoretical derivation process is as follows:

If the chest fluctuation caused by human respiration and heartbeat is simplified to sinusoidal vibration, the small amplitude change in the chest can be expressed as:

(1) $$g(t)=A_{r}\sin(2\pi f_{r}t)+A_{h}\sin(2\pi f_{h}t)$$

*A_r_* and *A_h_* are the amplitudes of the chest caused by human respiration and heartbeat, respectively. *f_r_* and *f_h_* are the frequencies of human respiration and heartbeat, respectively. The instantaneous distance between the radar and the surface of the human chest *d*(*t*) is equal to the sum of the average distance *d_0_* and the amplitude of the vibration of the chest *g*(*t*):

(2) $$d(t)=d_{0}+A_{r}\sin(2\pi f_{r}t)+A_{h}\sin(2\pi f_{h}t)$$

The impulse response of the radar channel is recorded as *h*(*τ,t*). Assuming that the environment remains stationary except for human chest fluctuations, the background signal can be viewed as a static function, and the periodic fluctuations in the chest behave as a periodic function in the impulse response [26]:

(3) $$h(\tau ,t)=a_{v}\delta (\tau -\tau_{v}(t))+\sum_{i}a_{i}\delta (\tau -\tau_{i})\;$$

*τ* is the sampling time for each pulse, also known as the fast time. *t* is the cumulative time that the radar emits multiple pulses, also known as slow time. *a_v_* is the amplitude of the reflection echo caused by chest fluctuations, *τ_v_*(*t*) is the time-delay change in the reflection echo caused by chest fluctuations. *a_i_* is the amplitude of the reflection echo caused by a stationary object in the environment, and *τ_i_* is the time delay of the reflection echo caused by a stationary object in the environment. *τ_v_*(*t*) may be further expressed as follows:

(4) $$\tau_{v}(t)=\frac{2d(t)}{v}=\frac{2(d_{0}+A_{r}\sin(2\pi f_{r}t)+A_{h}\sin(2\pi f_{h}t))}{v}=\tau_{0}+\tau_{r}\sin(2\pi f_{r}t)+\tau_{h}\sin(2\pi f_{h}t)$$

*v* is the propagation velocity of the electromagnetic wave, and the transmitted pulse signal of the radar is recorded as *s*(*t*). If the nonlinear factors such as pulse transmission distortion are not taken into account, the echo received by the receiver can be recorded as:

(5) $$r(\tau ,t)=s\left(t\right)*h(\tau ,t)=a_{v}s(\tau -\tau_{v}(t))+\sum_{i}a_{i}s(\tau -\tau_{i})$$

The echo signal is discretized and stored in a matrix. The total number of sampling points of single channel data is *M*, and the original echo matrix can be obtained by continuously recording *n*-channel Data:

(6) $$R_{M\times N}=r(m\delta_{s},nT_{s})=a_{v}s(m\delta_{s}-\tau_{v}(nT_{s}))+\sum_{i}a_{i}s(m\delta_{s}-\tau_{i})$$

*δ_s_* is the sampling interval, and *T_s_* is the time interval between the long-observed tracks. *m* = 1, 2, …, *M*, *n* = 1, 2, …, *N*.

## 3. Proposed Detection Method

**Figure 1:**
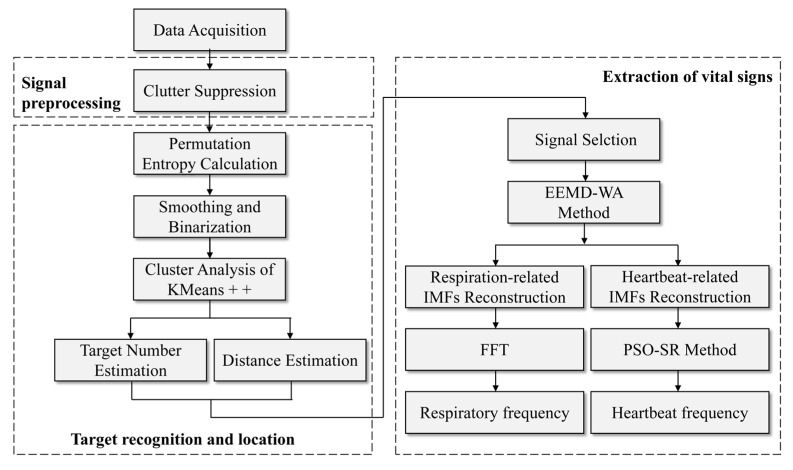
Specific flowchart of proposed method.

We propose a method based on the radar echo matrix to recognize, localize and extract the vital signs of multiple human targets. The flowchart of this method is shown in Figure 1. It includes three parts: signal preprocessing, target identification and location, and extraction of vital signs. Firstly, the time mean subtraction (TMS) method was used to filter out the static clutter in the echo signal. Then, according to the permutation entropy value of the slow time series corresponding to each distance gate, the human target area was initially screened. The distance gate labels were clustered using the one-dimensional K means++ algorithm to obtain the number and location of human targets in the environment. Then, the EEMD–WA method was applied to adaptively decompose the body surface vibration signal of each target into several intrinsic mode functions (IMFs) and obtain their main frequency. The IMFs whose main frequency is not in the respiratory and heartbeat frequency bands were removed as clutter. FFT processing was performed on the reconstructed respiratory signal to obtain respiratory frequency. The PSO–SR method was used for the reconstructed heartbeat signal to further extract the heartbeat frequency.

### 3.1. Signal Preprocessing

The time mean subtraction (TMS) method was used to filter the static clutter in the echo signal. First, all received pulses were averaged to obtain the reference received pulse, then the reference received pulse from each received pulse was subtracted to obtain the processed echo signal. The process was as follows:

(7) $$C[m]=\frac{1}{N}\sum_{i=1}^{N}R[m,i]$$

(8) R[m,n]=R[m,n]−C[m]

### 3.2. Target Recognition and Location

In order to recognize and locate trapped people in the environment, we developed a new method based on permutation entropy and the K means++ clustering algorithm. According to the permutation entropy value of the slow time series corresponding to each distance gate, the region of the human target was preliminarily screened. Using the one-dimensional K means++ algorithm to cluster the range gate tags, the number and location of human targets in the environment can be obtained.

**Figure 2:**
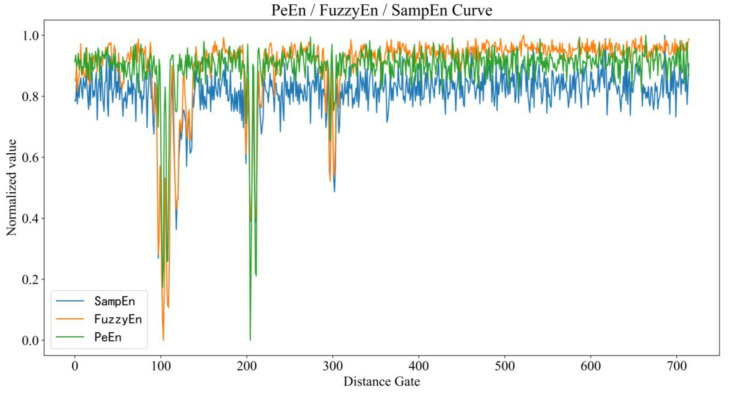
The curves of permutation entropy, sample entropy and fuzzy entropy when the three volunteers were 3, 6 and 9 m away from the radar.

Permutation entropy [27] is a parameter to measure the complexity of one-dimensional time series and is often used to detect the dynamic mutation of complex systems. Because the vibration of the human chest is periodic, the slow time direction data changes relatively regularly, and the permutation entropy value should be small, so it can be used for the positioning of human targets. When the three volunteers were 3 m, 6 m and 9 m away from the radar device, the results of calculating normalized permutation entropy, sample entropy [28] and fuzzy entropy [29] of radar echo data are shown in Figure 2. When the radar echo signal contains human vital signs, the three entropy values decrease significantly. The trailing phenomenon of the sample entropy and fuzzy entropy curves are more serious than permutation entropy. They are therefore not applicable to situations where the trapped persons are close to each other.

The permutation entropy curve was then smoothed and binarized. The smoothing process was as follows:

(9) S(m)=β⋅P(m)+(1−β)⋅S(m−1)

In the formula, *β* is the smoothing index, which is 0.9 in this study. *P*(*m*) is the original permutation entropy curve, and *S*(*m*) is the smoothed permutation entropy curve. The binarization process is as follows:

(10) $$B(m)=\{\begin{array}{cc} 0 & \left|S(m)-\max\left(S(m)\right)\right|\leq B_{r}\\ 1 & \left|S(m)-\max\left(S(m)\right)\right|>B_{r} \end{array}$$

(11) $$B_{r}=\frac{2}{M}\sum_{m=1}^{M}B(m)$$

If *B*(*m*) = 0, *m* = 1, 2, …, *M*, it was considered that there were no human targets in the environment. Otherwise, all points with a value of 1 were extracted to form a one-dimensional dataset and analyzed using the K means++ clustering algorithm [30]. The detailed procedure is described in reference [31]. The silhouette coefficient was used to evaluate the clustering effect and the number of clusters k corresponding to the largest silhouette coefficient was taken as the number of human targets in the environment. The distance gate corresponding to the minimum entropy value in each cluster was taken as the position of the human target *P_pos_*.

The actual distance between the radar and the human target can be expressed as:

(12) $$\mathrm{Distance}=\frac{v\times P_{pos}\times \delta_{s}}{2}$$

*v* is the propagation velocity of the electromagnetic wave, and in this paper the value is 3 × 10^8^ m/s.

### 3.3. Extraction of Vital Signs

#### 3.3.1. Noise Reduction in Life Signal

In order to solve the denoising problem in the feature extraction of life signals, an adaptive denoising method combining EEMD and WA was proposed. The method uses EEMD to decompose the body surface vibration signal into several intrinsic mode functions (IMF) adaptively and obtains the principal frequencies of each IMF by wavelet analysis. The IMF component, whose main frequency is not in the bands of breath and heartbeat frequency was filtered as clutter.

First, the slow time direction data of *P_pos_* was extracted as the vibration signal of the body surface and decomposed adaptively by the EEMD method. The EEMD method proposed by Huang [32] overcomes the mode aliasing caused by the traditional empirical mode decomposition (EMD) method. Due to the fact that white noise was distributed uniformly in the whole time–frequency space, by adding white noise to the original signal, the signal was continuous over different scales, which promotes the modal decomposition. The steps of the EEMD method were as follows:

Step 1. Normal white noise was added to the signal.

Step 2. The EMD method was used to decompose the signal with white noise into a series of intrinsic mode functions and residual signals adaptively:

(13) $$x(t)=\sum_{i=1}^{n}c_{i}(t)+r_{n}(t)$$

In the formula, *c_i_*(*t*) is the IMF component obtained by the *i*th screening, *n* is the number of the screening, and *r_n_*(*t*) is the residual component that cannot be decomposed further. Each IMF component corresponds to the local characteristic signals of different time scales in the original signal.

Step 3. Step 1 and step 2 were repeated and new white noise added with the same standard deviation each time.

Step 4. The IMF integration mean was taken as the final result.

In this paper, the complete ensemble number was set to 50. The standard deviation of white noise was 0.2 times the standard deviation of the original signal, and the stopping criterion of EMD decomposition was G. Riling criterion [33].

In order to filter out the IMF components without human life information, the wavelet variance of each IMF component was calculated. The wavelet variance reflects the distribution of fluctuating energy with frequency and can determine the main frequency components in a time series. The formula was as follows [34]:

(14) $$Var(a)=\int_{-\infty }^{+\infty }{\left|W_{f}(a,b)\right|}^{2}db$$

*W_f_*(*a*,*b*) is the wavelet transform coefficient. In this paper, complex Morlet wavelet [35] was chosen as the base wavelet function, and the frequency corresponding to the maximum wavelet variance was taken as the main frequency of each IMF.

Since the frequency range of the respiratory signal was 0.1 Hz~0.5 Hz and that of the heartbeat signal was 0.8 Hz~3.0 Hz, the IMFs whose main frequency does not belong to these two ranges were filtered, respectively.

#### 3.3.2. Extraction of Respiratory and Heartbeat Frequency

The FFT algorithm was used to analyze the reconstructed respiratory signal frequency domain, and the peak frequency was considered as the respiratory frequency of the human target.

**Figure 3:**
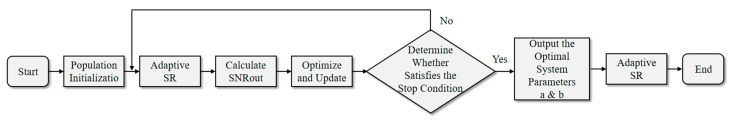
Detailed flow chart of adaptive stochastic resonance based on PSO.

The reconstructed heartbeat signal still has serious clutter interference, and the heartbeat frequency information cannot be extracted directly by FFT. Therefore, we proposed a new method for heartbeat frequency extraction based on PSO and variable-scale stochastic resonance. The reconstructed heartbeat signal was taken as the system input, and the signal-to-noise ratio of the system output signal was taken as the fitness function. The structural parameters of the stochastic resonance system were optimized by the PSO algorithm. By applying FFT and inverse scaling to the optimized stochastic resonance results, the heartbeat frequency of the human target was extracted. The detailed flow of this method is shown in Figure 3.

In a particular nonlinear system, the presence of noise can be combined with useful periodic signals to enhance the output of weak signals. At this time, the signal, noise and nonlinear stochastic system together produce a system phenomenon called stochastic resonance. This phenomenon can be used for blind periodic signal detection in low SNR scenarios [36,37] and has been widely used in the field of mechanical fault detection. Taking the classical bistable stochastic resonance system as an example, the expression is [38]:

(15) $$\frac{dx}{dt}=ax-bx^{3}+A\sin(2\pi ft+\varphi )+n(t)$$

*a* and *b* are the system parameters, both of which are greater than 0. *Asin*(2*πft* + *φ*) is the periodic signal to be measured, and *n*(*t*) is the additive white gaussian noise of intensity *D*. The potential function for this system is:

(16) $$U(x)=-\frac{a}{2}x^{2}+\frac{b}{4}x^{4}$$

**Figure 4:**
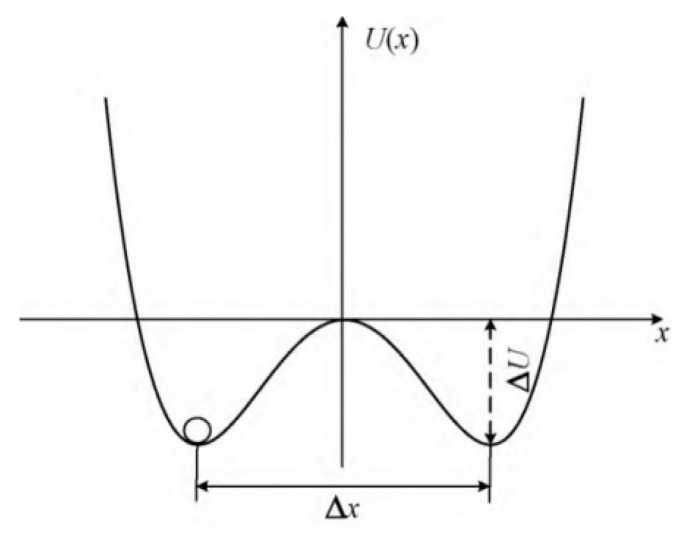
Potential function of bistable stochastic resonance system.

The system model is shown in Figure 4. The system has the properties of double wells, the potential well distance is $\Delta x=2\sqrt{\frac{a}{b}}$, the barrier height is $\Delta U=\frac{a^{2}}{4b}$. The essence is the motion state of the over-damped particle between two potential wells driven by periodic signal and noise. When the parameters *a*, *b* and *D* reach a certain matching state, the particles will jump back and forth in the two potential wells with the characteristic frequency of the input signal, that is, the stochastic resonance phenomenon occurs in the system.

Because of the limitation of adiabatic approximation theory, the above method is only suitable when the signal amplitude, frequency and noise intensity are far less than 1. In order to solve this problem, we designed a linear transformation *y*(*τ*) = *x*(*t*), *τ* = *pt* and inserted it into the Formula (15):

(17) $$p\cdot \frac{dy}{d\tau }=ay-by^{3}+A\sin(2\pi f\frac{\tau }{p})+n(\frac{\tau }{p})$$

*p* is the transformation coefficient, which is greater than 1. The above formula can be further simplified to:

(18) $$\frac{dy}{d\tau }=\frac{a}{p}y-\frac{b}{p}y^{3}+\frac{A}{p}\sin(2\pi f\frac{\tau }{p})+\frac{1}{p}n(\frac{\tau }{p})$$

To make $a1=\frac{a}{p}$, $b1=\frac{b}{p}$, $f1=\frac{f}{p}$, the above formula can be rewritten as:

(19) $$\frac{dy}{d\tau }=a_{1}y-b_{1}y^{3}+\frac{A}{p}\sin(2\pi f_{1}\tau )+\frac{1}{p}n(\frac{\tau }{p})$$

Through scale transformation, the target parameters *a*_1_, *b*_1_, *f*_1_ are all small parameters. The amplitude of signal and noise is reduced to $\frac{1}{p}$ of the original signal. However, the essential meaning of each part of the equation is not changed, so the detection of large parameter signals can be realized.

In order to realize synchronous optimization of system parameters *a* and *b*, the Particle Swarm Optimization (PSO) algorithm [39] was introduced into the bistable SR system. The fourth-order Runge–Kuta algorithm was used to solve the bistable model [40]. The fitness function selected the output SNR of the bistable system, which is defined as the ratio of the energy at the characteristic frequency of the output signal to the noise energy at a frequency near the characteristic frequency.

When the stochastic resonance effect of the system was optimal, the period component of the output waveform in the time domain was enhanced, and the amplitude of the characteristic frequency was prominent. The human heartbeat frequency could be obtained by inverse scaling of the peak frequency of the system output signal.

## 4. Experiments

### 4.1. Construction of the UWB Radar System

**Figure 5:**
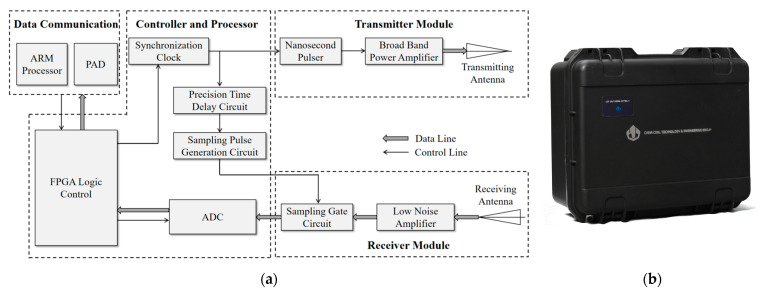
(**a**) Diagram of the UWB radar system (**b**) Self-developed experimental UWB radar system.

The experiment used a self-developed UWB radar system. The system consisted of four parts: transmitter module, receiver module, controller and processor module, and data communication module. In this system, the transmitter module adopted a high-stability, high-power pulse source based on a field effect tube. The center frequency of the transmitted pulse signal was 400 MHz ± 20 MHz, and the pulse width was 2 nanoseconds. The receiving module adopted a high-speed equivalent sampling method, and the sampling process was realized by a sampling gate circuit. The equivalent sampling frequency was 5 GSa/s, and the receiving sensitivity was −60 dbm. The transceiver antenna adopted an improved butterfly antenna, which had the advantages of simple structure, light weight and good radiation characteristics. The length of the antenna was 400 mm, and the width was 130 mm. The opening angle was 60°. A metal shielding cavity was designed to suppress the backward radiation of the antenna. By loading the absorbing material in the shielding cavity, the scattering caused by the residual current at the end of the antenna was suppressed, and the direct coupling signal between the transmitting antenna and the receiving antenna was reduced. The master control system based on FPGA could realize timing control, especially picosecond-level precise delay sequential logic control of equivalent sampling, and data processing functions. In the data communication module, the ARM processor establishes a wireless data communication network through the WiFi module and PAD and could export the data stored in the FPGA for subsequent analysis in this paper. The block diagram of the UWB radar system is shown in Figure 5.

The main working process of the system is as follows: the transmitter and the transmitting antenna were utilized to transmit ultra-wideband nanosecond electromagnetic wave pulses to the detection area. The ultra-wideband electromagnetic wave pulse generated reflected echoes after encountering human targets. The receiving antenna array received the echo of the human body target. After high-speed random equivalent sampling, it entered the ADC to convert it into a digital signal, and then stored it using the FPGA. During the experiment, the sampling frequency of the radar system was set to 39 GHz in the fast time domain and 8 Hz in the slow time domain. The main parameters are shown in Table 1.

### 4.2. Experimental Scheme Design

**Figure 6:**
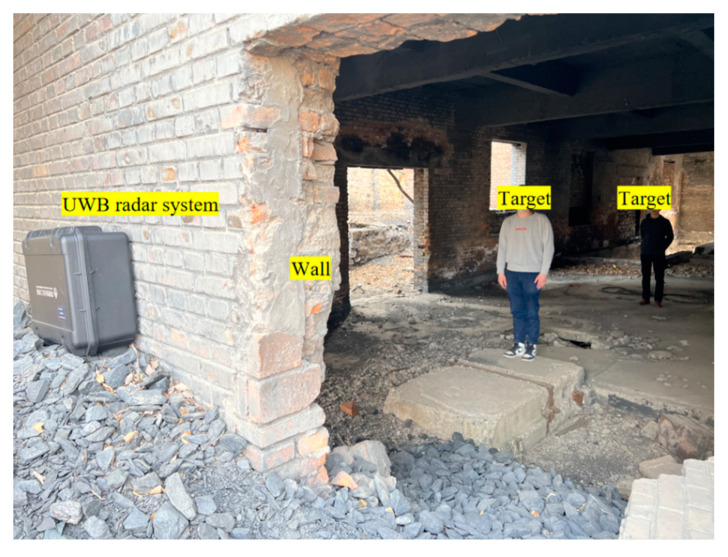
Photo of the experiment site.

In order to test the effectiveness of the proposed method in the practical sense, we carried out a series of experiments using a self-developed UWB radar system in an abandoned factory building on the outskirts of Beijing, China. In the first experiment, the two volunteers were at a distance of 5 m and 10 m from the radar. In the second experiment, the two volunteers were close to each other, at a distance of 5 m and 6 m from the radar. The third experiment involved three volunteers at distances of 3 m, 6 m and 9 m from the radar. Table 2 shows the basic information of the volunteers who participated in the experiment. During the experiment, the radar signal penetrated a 30 cm brick wall, and the volunteers stood still against the radar antenna and breathed normally. The different targets were staggered along the radar azimuth dimension. At the same time, a contact breathing belt and a pulse sensor were used to measure the frequency of breathing and heartbeat as a reference. Figure 6 is a photo taken at the experimental site.

## 5. Results and Discussion

### 5.1. Performance of the Signal Preprocessing Method

**Figure 7:**
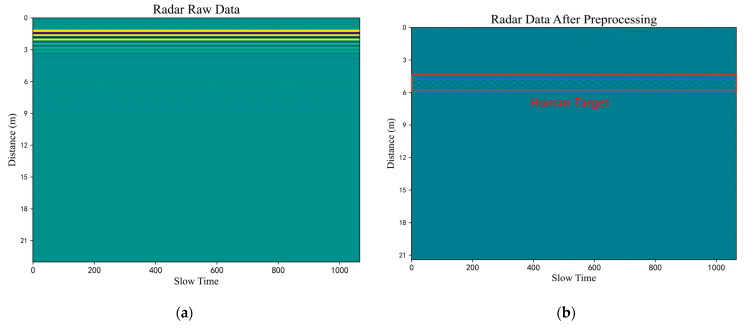
In the first experiment, when the two volunteers were 5 and 10 m away from the radar: (**a**) Raw radar data (**b**) Radar data after preprocessing.

Using the first experiment as an example, the results of the method were analyzed in detail. Figure 7a shows the original radar echo matrix. As can be seen from the figure, the signal containing human information was almost completely covered by direct wave and clutter signals. Figure 7b shows the radar echo matrix after preprocessing using the time mean subtraction method. It can be seen that the direct wave closer to the radar device was effectively weakened and the life signal was enhanced. A weak periodic fluctuation of the slow time series can be observed at a distance of around 5 m, where the human target is located. However, the other human target which is far away from the radar device still cannot be identified and needs further processing.

### 5.2. Performance of Target Recognition and Location Method

**Figure 8:**
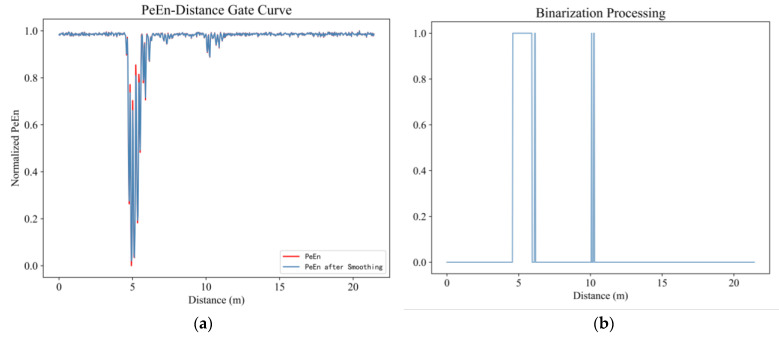
In the first experiment, when the two volunteers were 5 and 10 m away from the radar: (**a**) The PE curve before and after smoothing (**b**) Results after binary processing.

**Figure 9:**
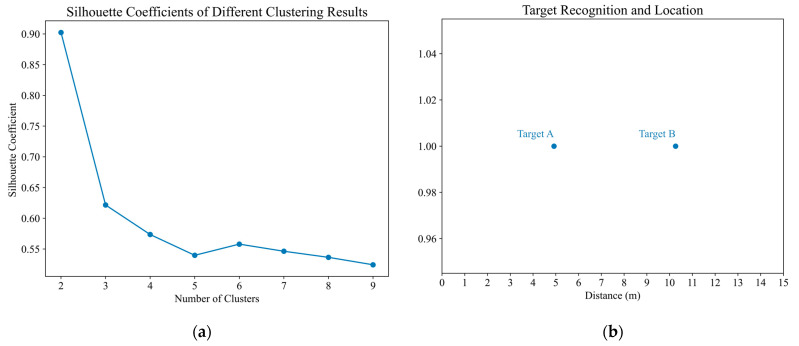
In the first experiment, when the two volunteers were 5 and 10 m away from the radar: (**a**) Results of clustering performance evaluation (**b**) Results of target recognition and location.

As shown in Figure 8, the permutation entropy curve is smoothed and binarized. Then, the binarization points with a value of 1 were extracted to form the data set numbered by distance gate, and the number of clusters as a variable parameter is selected to cluster the data set using the one-dimensional K means++ method. The effect of clustering was evaluated by the silhouette coefficient, and the number of clusters with the maximum silhouette coefficient were considered to be the number of human targets. It can be seen from Figure 9a that the value of the silhouette coefficient was the largest when the number of clusters was two, so it can be considered that there were two human targets in the detection area. The actual distance between the radar and each human target was estimated according to Formula (12). Figure 9b shows the final detection results. The distance between target A and the radar device was 4.92 m, and the relative error was 1.6%. Target B was 10.26 m away from the radar device, and the relative error with the true value was 2.6%.

### 5.3. Performance of Vital Signs Information Extraction Method

**Figure 10:**
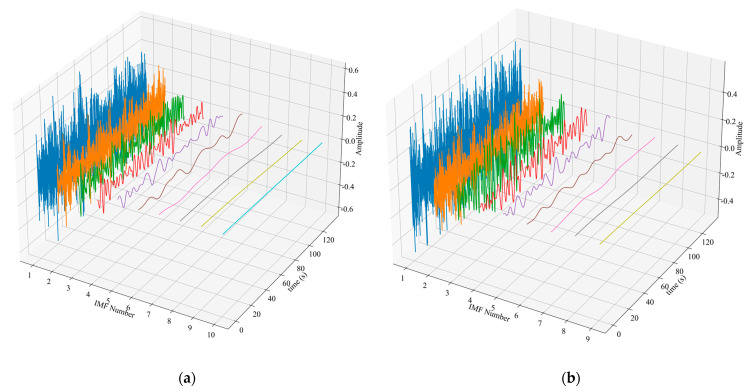
In the first experiment, when the two volunteers were 5 and 10 m away from the radar: EEMD decomposition results (**a**) Target A (**b**) Target B.

**Figure 11:**
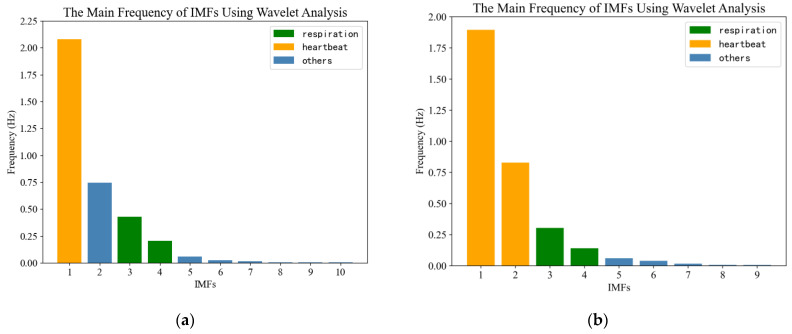
In the first experiment, when the two volunteers were 5 and 10 m away from the radar: The main frequency of each IMF (**a**) Target A (**b**) Target B.

**Figure 12:**
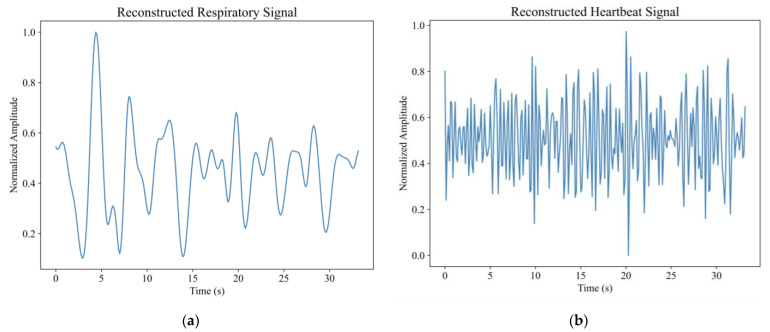

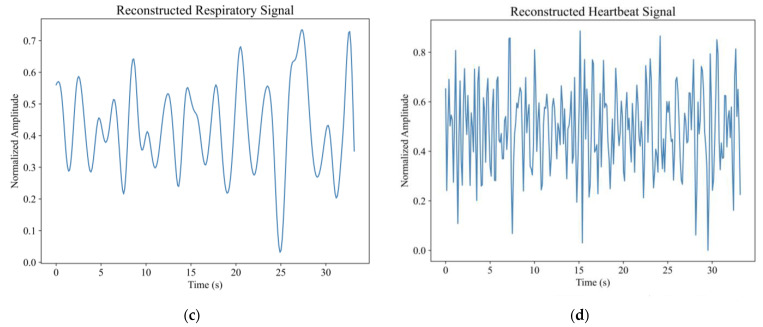
In the first experiment, when the two volunteers were 5 and 10 m away from the radar: (**a**) Reconstructed respiratory signal of target A (**b**) Reconstructed heartbeat signal of target A (**c**) Reconstructed respiratory signal of target B (**d**) Reconstructed heartbeat signal of target B.

The slow time series data of the position of the human target *P_pos_* were extracted for further analysis. The echo signal of the human target was decomposed into several IMF components by the EEMD algorithm, as shown in Figure 10. The main frequencies of the IMFs were calculated and plotted in Figure 11. As can be seen from Figure 11a, the respiratory signal of target A is reconstructed by IMF3 and IMF4, and the heartbeat signal is reconstructed by IMF1. As can be seen from Figure 11b, the respiratory signal of target B is reconstructed by IMF3 and IMF4, and the heartbeat signal is reconstructed by IMF1 and IMF2. The results are shown in Figure 12.

**Figure 13:**
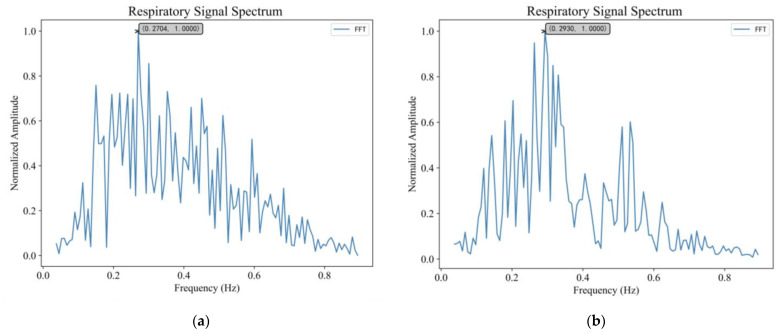
In the first experiment, when the two volunteers were 5 and 10 m away from the radar: (**a**) Respiratory signal spectrum of target A (**b**) Respiratory signal spectrum of target B.

The respiratory signal of target A and B were analyzed in the frequency domain using FFT and the results are shown in Figure 13. As can be seen from Figure 13a, the main peak frequency extracted by the FFT method was 0.2704 Hz. The measured respiratory frequency of human target A was 0.28 Hz, and the relative error was 3.4%. As can be seen from Figure 13b, the respiratory frequency extracted by FFT was 0.2930 Hz, and the measured respiratory frequency of target B was 0.32 Hz. The relative error was 8.4%.

**Figure 14:**
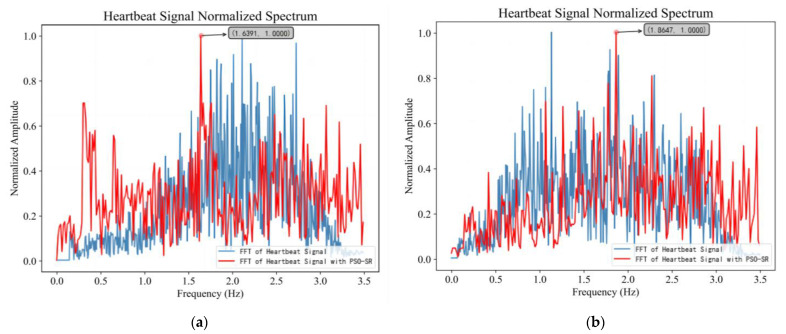
In the first experiment, when the two volunteers were 5 and 10 m away from the radar: (**a**) Heartbeat signal spectrum of target A (**b**) Heartbeat signal spectrum of target B.

The PSO-SR method was used to further obtain the heartbeat frequency of the human target. The particle population size was set to 100, and the maximum number of iterations was 500. Shown in Figure 14a,b are the results of heartbeat signal processing for targets A and B, respectively. As can be seen from Figure 14a, there was still a large amount of noise in the input signal and the frequency of the heartbeat could not be extracted by FFT. After adaptive stochastic resonance processing, the spectrum of the output signal was very high and prominent at 1.6391 Hz. The measured value of heartbeat frequency of target A is 1.53 Hz, and the relative error was 7.1%. It can be seen that the heartbeat frequency extraction method based on PSO-SR can extract the heartbeat frequency of the human target effectively in the strong noise background, and improve the SNR of the output signal. As can be seen from Figure 14b, the heartbeat frequency of target B was 1.8647 Hz with an error of 11.7%.

### 5.4. Further Verification of Performance

The second experiment was to verify the performance of the method when the targets were close to each other. The third experiment was to verify the performance of the method when the number of targets in the environment was higher. Table 3 and Table 4 summarize the results of the three experiments. As can be seen from Table 3, in the multi-target detection scenarios, the proposed method could successfully identify each human target in the environment and provide accurate location information. The average relative error of the distance measured was 1.83%. In the second experiment, even if two targets were close to each other, this method can effectively distinguish and successfully extract the information from the distant target which is interfered with by the former target.

As can be seen from Table 4, the average relative error of the respiratory frequency was 4.27%, and the average relative error of the heartbeat frequency was 6.23%. When the volunteers are farther from the radar, the accuracy of the extraction of breathing and heartbeat frequency decreased. Combined with the spectrum of the heartbeat signal shown in Figure 14, it can be seen that this method can improve the success rate and accuracy of heartbeat frequency extraction in multi-target scenes compared with the traditional FFT algorithm.

## 6. Conclusions

Life detection technology based on UWB radar can be used for the search and rescue of survivors. When there are multiple human targets to be detected in the environment and the noise interference is severe, it is difficult for the existing echo signal processing methods to accurately recognize and locate each target and simultaneously extract breathing and heartbeat information. Therefore, this paper proposes a multi-target localization and vital sign extraction method using ultra-wide band radar. Analysis of the results leads to the following conclusions:

(1) The target recognition and positioning method based on permutation entropy and K means++ clustering can successfully recognize multiple human targets in the environment, and accurately extract the location information of distant targets interfered with by the former targets. The average relative error of the distance measured by the method was 1.83%.
(2) In this paper, an adaptive denoising method for vital signs extraction based on EEMD–WA was proposed, which could effectively filter the clutter signal and reconstruct the breathing and heartbeat signals of human targets. The respiratory frequency was obtained by FFT of the reconstructed respiratory signal, and the average relative error was 4.27%.
(3) In order to solve the problem that existing methods cannot effectively extract heartbeat information, this paper proposed a heartbeat frequency extraction method based on PSO-SR, which can successfully extract the heartbeat frequency of each target in the environment, and the average relative error was 6.23%.

In future research work, further optimization of the method in this paper, and the conduction of a large number of experimental verifications to further improve the accuracy of detection and to shorten the time required for detection are proposed. In addition, the situation where multiple human targets are distributed at the same distance will be further considered to expand the scope of application of the method.

## Figures and Tables

**Table 1:** Parameters of UWB radar system.

Parameter	Numerical Value
Center frequency	400 MHz ± 20 MHz
Pulse width	2 ns
Fast time domain sampling frequency	39 GHz
Slow time domain sampling frequency	8 Hz
Reception sensitivity	−60 dBm
Equivalent sampling rate	5 G Sa/s

**Table 2:** Experimental scheme settings.

No.	Volunteer	Gender	Height (cm)	Weight (kg)	Distance (m)
1	A	male	176	70	5.0
	B	male	179	75	10.0
2	A	male	176	70	5.0
	C	female	160	51	6.0
3	A	male	176	70	3.0
	C	female	179	51	6.0
	B	male	160	75	9.0

**Table 3:** Results and performance of the target recognition and location algorithm.

No.	Volunteer	Target Number		Position (m)		
		Result	Truth Value	Result	Truth Value	Relative Error
1.	A	2	2	4.92	5.00	1.6%
	B			10.26	10.00	2.6%
2.	A	2	2	5.13	5.00	2.6%
	B			5.94	6.00	1.0%
3.	A	3	3	3.06	3.00	2.0%
	C			6.12	6.00	2.0%
	B			8.91	9.00	1.0%

**Table 4:** Results and performance of the extraction algorithm of vital signs.

No.	Volunteer	Respiratory Frequency (Hz)			Heartbeat Frequency (Hz)		
		Result	Truth Value	Relative Error	Result	Truth Value	Relative Error
1.	A	0.2704	0.28	3.4%	1.6391	1.53	7.1%
	B	0.2930	0.32	8.4%	1.8647	1.67	11.7%
2.	A	0.2929	0.31	5.5%	1.4862	1.42	4.7%
	B	0.3358	0.33	1.8%	1.4281	1.41	1.3%
3.	A	0.2897	0.29	0.1%	1.4603	1.40	4.3%
	C	0.2739	0.29	5.6%	1.4921	1.42	5.1%
	B	0.2846	0.30	5.1%	1.7724	1.62	9.4%

## Data Availability

Not applicable.
